# Characterisation of Ovarian Cancer Cell Line NIH-OVCAR3 and Implications of Genomic, Transcriptomic, Proteomic and Functional DNA Damage Response Biomarkers for Therapeutic Targeting

**DOI:** 10.3390/cancers12071939

**Published:** 2020-07-17

**Authors:** Alice Bradbury, Rachel O’Donnell, Yvette Drew, Nicola J. Curtin, Sweta Sharma Saha

**Affiliations:** 1 Newcastle Centre for Cancer, Translational and Clinical Research Institute, Faculty of Medical Sciences, Newcastle University, Newcastle upon Tyne NE2 4HH, UK; a.bradbury@newcastle.ac.uk (A.B.); yvette.drew@newcastle.ac.uk (Y.D.); nicola.curtin@newcastle.ac.uk (N.J.C.); 2 Northern Cancer Alliance, Northern Centre for Gynaecological Surgery, Newcastle Hospitals NHS Foundation Trust, Newcastle upon Tyne NE1 4LP, UK; 3 Northern Centre for Cancer Care (NCCC), Newcastle Hospitals NHS Foundation Trust, Newcastle upon Tyne NE7 7DN, UK

**Keywords:** ovarian cancer, PARP, ATR, platinum, homologous recombination repair, non-homologous end-joining

## Abstract

In order to be effective models to identify biomarkers of chemotherapy response, cancer cell lines require thorough characterization. In this study, we characterised the widely used high grade serous ovarian cancer (HGSOC) cell line NIH-OVCAR3 using bioinformatics, cytotoxicity assays and molecular/functional analyses of DNA damage response (DDR) pathways in comparison to an ovarian cancer cell line panel. Bioinformatic analysis confirmed the HGSOC-like features of NIH-OVCAR3, including low mutation frequency, TP53 loss and high copy number alteration frequency similar to 201 HGSOCs analysed (TCGA). Cytotoxicity assays were performed for the standard of care chemotherapy, carboplatin, and DDR targeting drugs: rucaparib (a PARP inhibitor) and VE-821 (an ATR inhibitor). Interestingly, NIH-OVCAR3 cells showed sensitivity to carboplatin and rucaparib which was explained by functional loss of homologous recombination repair (HRR) identified by plasmid re-joining assay, despite the ability to form RAD51 foci and absence of mutations in HRR genes. NIH-OVCAR3 cells also showed high non-homologous end joining activity, which may contribute to HRR loss and along with genomic amplification in ATR and TOPBP1, could explain the resistance to VE-821. In summary, NIH-OVCAR3 cells highlight the complexity of HGSOCs and that genomic or functional characterization alone might not be enough to predict/explain chemotherapy response.

## 1. Introduction

Genomic instability, a key enabling characteristic of cancer [1], is often caused by defects in DNA damage response (DDR) pathways. DDR defects can however be exploited therapeutically in two ways: (i) using radiation/chemotherapy to cause genotoxic damage that cannot be effectively resolved because of the DDR defect, or (ii) by targeting a complementary DDR pathway, such that endogenous DNA damage cannot be repaired, resulting in “synthetic lethality” [2]. The prime example of this is the synthetically lethal use of poly (ADP-ribose) polymerase (PARP) inhibitors (PARPi), that block DNA single strand break repair, in cells with defective homologous recombination DNA repair (HRR). This principle has been successfully applied clinically not only in HGSOC, where HRR defects are observed in ~50%, but also in breast, pancreatic and prostate cancer [3] with HRR defects due to BRCA1/2 mutation.

High grade serous ovarian cancer (HGSOC) is the most common histological subtype of epithelial ovarian cancer (EOC), and is characterised by p53 loss in ~96% of cases accompanied by a high frequency of copy number alterations (CNAs) [4]. Despite low rates of mutation frequencies, HGSOCs often carry mutations in genes involved in the DDR, with BRCA mutations affecting HRR being well described [4]. In 2018, HGSOC accounted for over 280,000 new cancer cases globally and is the leading cause of death from gynaecological malignancy [5] with a poor 5-year survival rate of around 45% (World Cancer Coalition Atlas, 2018). The approval of PARPi in women with HGSOC harbouring HRR defects, and as maintenance therapy in women with platinum sensitive tumours has resulted in significant clinical benefits with prolongation of disease free survival irrespective of BRCA mutation or HRD status [6,7], as well as improved overall survival in platinum-sensitive cases [8]. Despite the addition of PARPi and bevacizumab [9], alongside best standard of care involving surgical cytoreduction and platinum-based systemic therapy, there is an apparent need to optimise existing therapies and develop new therapeutic strategies.

Studies are underway to identify determinants of sensitivity to existing chemotherapies [10], and small molecule inhibitors targeting several other DDR pathway molecules (ATM, DNA-PK, CHK1, CHK2, WEE1 and ATR) are being explored pre-clinically and clinically [11,12,13,14]. Cell lines are useful models to explore determinants of sensitivity to cytotoxic drugs as well as novel synthetic lethalities. Isogenically matched cell line pairs can identify if a single gene confers resistance/sensitivity [15], whilst panels of cell lines can indicate molecular characteristics associated with drug response [16,17]. However, these approaches may show discordance in their findings, hence a molecular analysis alone may not be sufficient to accurately predict an individual clinical response [18,19].

In this study, we investigated one of the most widely studied ovarian cancer cell line, NIH-OVCAR3, as a model of HGSOC, established in 1982 from ascites from a progressive ovarian adenocarcinoma that was resistant to cyclophosphamide, cisplatin and doxorubicin [20]. Molecular analyses [10,21,22] has confirmed the cells as representative of HGSOC and have been studied as ovarian cancer model with ~2000 PubMed publications. Being an extensively used cell line, NIH-OVCAR3 was also included in the NIH NCI-60 cell line panel study (https://dtp.cancer.gov/discovery_development/nci-60/), as well as large scale genomic and drug sensitivity studies including Cancer Cell Line Encyclopedia (CCLE) led by the Broad Institute (https://portals.broadinstitute.org/ccle, funded by Novartis), and the Genomics of Drug Sensitivity in Cancer (GDSC) study led by the UK Wellcome Sanger Institute (https://www.cancerrxgene.org/).

Our study confirmed NIH-OVCAR3 cells to be highly representative of HGSOCs with functional loss of p53 and a very high number of CNAs using published genomic data (CCLE). However, among a panel of ovarian cancer cell lines analysed, the NIH-OVCAR3 cells exhibited an unexpected drug response profile, which was explored further using genomic, proteomic and functional approaches. NIH-OVCAR3 cells were found to be resistant to the ATR inhibitor (ATRi), VE-821, but as sensitive to the PARPi, rucaparib, and carboplatin as cell lines with known *BRCA* mutations. NIH-OVCAR3 showed no functionally inactivating mutations in HRR genes and were competent in forming RAD51 foci in response to DNA damage induction (an accepted biomarker of HRR function). However, further analysis confirmed functional loss of HRR, likely downstream of RAD51, and concomitant activation of the non-homologous end joining (NHEJ) pathway of double strand break (DSB) repair. Genomic analysis identified amplifications in several DDR genes including ATR and TOPBP1 which, along with high NHEJ activity, may explain the relative resistance to VE-821. Additionally, when DDR genes with alterations (genomic and protein level) in NIH-OVCAR3 cells were analysed within the HGSOCs in the TCGA study, similar alterations in genes including ATR, TOPBP1 and XRCC6 (Ku70) were frequently observed in HGSOCs and are hence likely to be clinically relevant biomarkers of response. In summary, analysis of NIH-OVCAR3 highlights the complexity of the molecular profile of ovarian cancer and the difficulties in predicting sensitivity using a single or even multiple molecular determinants.

## 2. Results

### 2.1. NIH-OVCAR3 Cells Are Representative of HGSOC, with TP53 Mutation, a Low Number of Mutations and High Frequency of Copy Number Alterations

**Figure 1:**
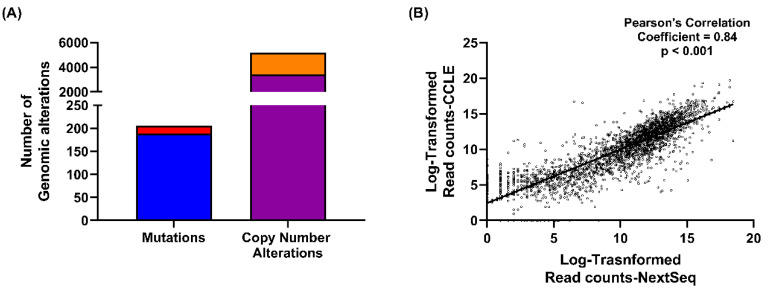
Confirmation of the NIH-OVCAR3 cells as representative of HGSOC. (**A**) Mutation and copy number alteration data from the CCLE database was analysed. Mutations were categorised as homozygous (**red**) or heterozygous (**blue**) using a variant allele frequency cut-off of 0.8, mutations with variant allele frequency >0.8 were considered homozygous. The copy number alterations were categorised using their GISTIC scores of −2 or 2 (amplifications: GISTIC score of 2 (**orange**) and deletions: GISTIC score of −2 (**purple**). (**B**) RNA-seq read counts of 2426 genes from the CCLE database were correlated with the read counts for the same 2426 genes established from targeted RNA-sequencing to confirm authenticity of the NIH-OVCAR3 cells in our possession.

Analysis of the genomic profile of NIH-OVCAR3 cells (CCLE database) confirmed the previous characterisation as being representative of HGSOC [21] with a low frequency of mutations and a high frequency of CNAs (Figure 1A). Of the 205 mutations for which allele frequency data was available, only 17 were homozygous mutations (Variant allele frequency ≥ 0.8). 5191 CNAs were reported in the NIH-OVCAR3 cells, of which 1787 were genomic amplifications and 3404 were deep deletions. Further analysis of 120 DDR genes identified homozygous mutation in only one gene, TP53, a common feature of HGSOC. Amplifications were found in 12 DDR genes: TOPBP1, XRCC3, PARP9, PARP15, PARP14, PARP2, APEX1, POLB, MBD4, POLE2, ATR and EME2 and deep deletions were seen in 19 DDR genes: TP53, BRCA2, RAD51D, FANCA, FANCI, RPA1, RPA2, BLM, PALB2, TOP2A, XRCC6, PARP4, LIG3, POLG, NEIL1, ATM, CHEK1, ERCC4 and PCNA.

To confirm the authenticity of the NIH-OVCAR3 cells used in this study, the cells were authenticated by STR profiling and molecular analysis using targeted RNA-seq. The RNA-seq data of 2426 genes analysed using the HTG Oncology Biomarker panel was correlated with RNA-Seq data downloaded from the CCLE database. There was a strong positive correlation (*r*-value = 0.84) between the two datasets (Figure 1B) which confirmed NIH-OVCAR3 cells to be typical of those previously characterised in the literature.

### 2.2. NIH-OVCAR3 Cells Are Resistant to ATR Inhibitor VE-821, but Sensitive to Carboplatin and the PARP Inhibitor, Rucaparib

**Figure 2:**
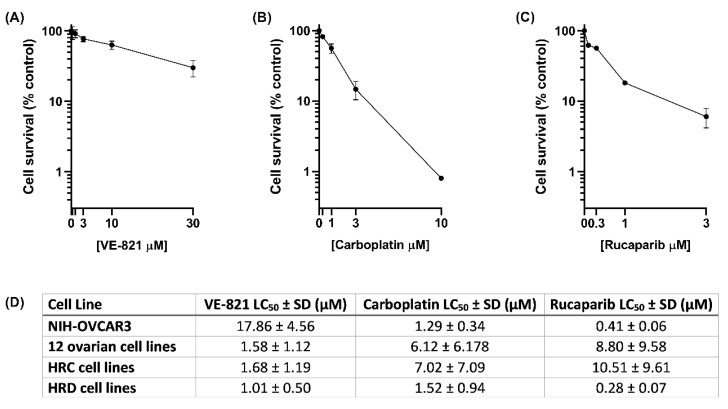
Sensitivity of NIH-OVCAR3 cells to VE-821, carboplatin and rucaparib. NIH-OVCAR3 cells were exposed to (**A**) VE-821 for 48 h, (**B**) carboplatin for 24 h or (**C**) rucaparib for 24 h. Media was replaced with drug free media for 21 days to allow colony formation. Data are the mean and standard deviation of at least three independent experiments. (**D**) Sensitivity of an additional 12 ovarian cancer cell lines to the three drugs was calculated in the same way and LC_50_ values were calculated. Sensitivity of NIH-OVCAR3 cells in comparison to the mean of 12 other ovarian cancer cell lines, and the cell lines grouped by their HRR status into HRR competent (HRC, 3 cell lines) or HRR defective (HRD, 2 cell lines). Data are pooled from 3 independent experiments per cell line. Western blot confirming the loss of TP53 function in NIH-OVCAR3 cells can be found at Figure S3.

One of the key roles of ATR is signalling replication stress to the S and G2/M checkpoints to pause the cell cycle allowing for DNA repair. Loss of G1 checkpoint control in cancer, by functional loss of TP53, for example, is predicted to result in cancer cells more likely to enter S phase with increased replication stress and an increased reliance on the S and G2/M checkpoints and hence ATR function. Given the high frequency of functionally inactivating TP53 homozygous mutations among HGSOCs, ATR inhibition was hypothesised to be an attractive therapy. However, despite TP53 mutation in the NIH-OVCAR3 cells (Figure 1A), they were 11-fold more resistant to the ATRi, VE-821, compared to a panel of 12 ovarian cancer cell lines (Figure 2A,D).

Originating from a tumour clinically refractive to cyclophosphamide, cisplatin and doxorubicin, in original publications the NIH-OVCAR3 cells were reported to be platinum resistant [20,23]. However, more recent studies such as Beaufort et al., 2014 [10] and the GDSC database (https://www.cancerrxgene.org/) highlight conflicting results. We found NIH-OVCAR3 cells were 5-fold more sensitive to carboplatin than the 12 ovarian cancer cell lines (Figure 2B,D), which was comparable to the ovarian cancer cells with homozygous mutations in BRCA1. NIH-OVCAR3 cells were also 4-fold more sensitive to cisplatin (LC_50_ 0.18 µM) compared to 2 representative ovarian cancer cell lines (IGROV1 and CAOV3) in the panel (mean LC_50_ 0.70 µM) (Table S1 and Figure S1) confirming the platinum sensitivity of NIH-OVCAR3.

Because of the similar carboplatin sensitivity of NIH-OVCAR3 cells to that of the *BRCA* mutant cells, their sensitivity to the PARPi, rucaparib was also investigated (Figure 2C). NIH-OVCAR3 cells were 21-fold more sensitive to rucaparib than the 12 other cell lines collectively, 26-fold more sensitive than HRR competent (HRC) cell lines (COV318, CAOV3, ES-2, OAW42, A2780, CP70-B1, CP70-A2, IGROV1, UWB1.289 + Brca1, NUCOLL43) with a similar level of sensitivity to the 2 *BRCA1* mutant HRR defective (HRD) cell lines (COV362 and UWB1.289) [24], suggesting a defect in HRR (Figure 2D). The NIH-OVCAR3 cells did not carry any mutations in known HRR genes, but deep deletions were reported in several HRR-related genes including BRCA2. However, the BRCA2 gene expression in the NIH-OVCAR3 cells (normalised mRNA expression value = 0.71) was similar to that seen in the 12 ovarian cancer cell lines (normalised mRNA expression value = 0.74). We speculate an unlikely impact on gene function as although genomic deletions identified by CNAs may cause reduced expression, they rarely result in complete loss of protein expression [25]. Cytotoxicity analysis with other drugs including doxorubicin, gemcitabine, paclitaxel and topotecan identified similar sensitivities of NIH-OVCAR3 cells to the rest of the 12 cell lines (Table S2), highlighting that the cells responded differently specifically to platinum agents, PARPi (rucaparib) and ATRi (VE-821), which required further exploration.

### 2.3. NIH-OVCAR3 Cells Are Resistant to VE-821 Despite Functional Loss of p53 and Significant ATR Inhibition

Loss of p53 function has been considered one of the major determinants of sensitivity to ATR inhibition, however, the NIH-OVCAR3 cells were found to be resistant to VE-821 despite functionally inactivating homozygous mutation in TP53 together with deep deletion. Therefore, we sought to determine if p53 function had been restored in these cells. Western blot analysis revealed that despite deep deletion reported in TP53, the NIH-OVCAR3 cells still expressed p53 protein, confirming our speculation that genomic deletions are unlikely to cause complete loss of protein expression. However, when treated with Nutlin-3, an MDM2 antagonist that inhibits MDM2-p53 interaction resulting in p53 activation, there was no induction of either MDM2 or p21 (indicators of p53 activation) in NIH-OVCAR3 compared to a p53 wild type (WT) cell line, confirming functional loss of p53 caused by the mutations (Figure S2).

**Figure 3:**
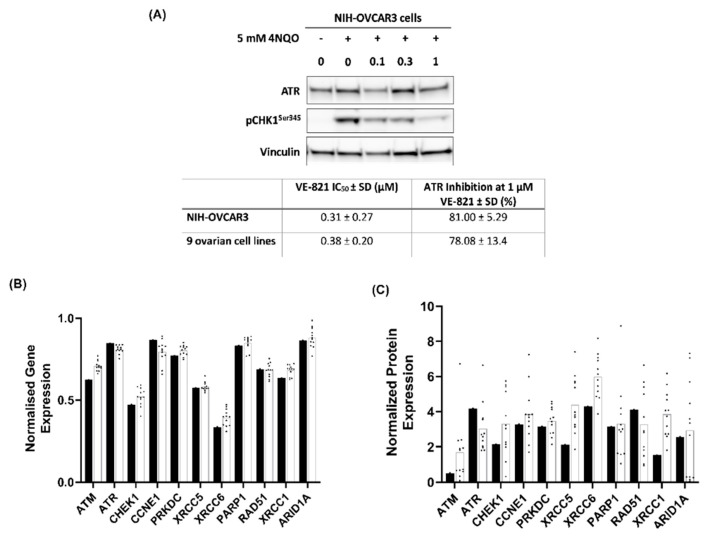
Inhibition of ATR by VE-821 and the expression of potential determinants of sensitivity and resistance to VE-821 in the NIH-OVCAR3 cells. (**A**) NIH-OVCAR3 cells and 9 other ovarian cancer cell lines were exposed to 4NQO ± VE-821 as indicated for 1 hr before western blotting. Chemiluminescence was quantified using Syngene software and expression of pCHK1Ser345 was normalised to vinculin expression. Western blot image is from a single representative experiment with pooled data from 3 independent experiments per cell line used to calculate the mean VE-821 IC50 and percentage inhibition at 1 µM VE-821. (**B**) Bar chart represents log-transformed read counts obtained from RNA-Seq data for each gene normalized to ACTB mRNA expression for NIH-OVCAR3 cells (**black bars**) and the mean mRNA expression of each gene across 12 cell lines (**white bars**) where each black dot represents one cell line. (**C**) Bar chart represents protein expression normalised to Ponceau-s staining calculated using ImageJ software in NIH-OVCAR3 cells (black bars) and the mean protein expression of each gene across 12 cell lines (**white bars**) where each black dot represents one cell line. Data are from a single experimental repeat. [PRKDC: DNA-PKcs, XRCC5: Ku80, XRCC6: Ku70]. Western blot of ATR activation and inhibition by 4NQO ± VE-821 in NIH-OVCAR3 cells can be found at Figure S4. Western blot of baseline protein expression in panel of ovarian cancer cell lines can be found at Figure S5.

To exclude the possibility that the resistance to VE-821 observed in NIH-OVCAR3 cells was due to VE-821 failing to inhibit ATR in these cells, we measured induction of ATR activity by 4NQO (a DNA damaging agent and inducer of ATR) [26] and its inhibition by VE-821 using pCHK1^Ser345^, a specific marker of ATR activity [27]. The IC_50_ and the percentage ATR inhibition at 1 µM VE-821 for the NIH-OVCAR3 cells and nine other cell lines analysed was found to be similar (Figure 3A) indicating that resistance was not due to lack of ATR inhibition.

Next, we investigated expression of 11 DDR proteins that have previously been identified as determinants of sensitivity to ATR inhibition [28]. The normalised mRNA expression in the NIH-OVCAR3 cells and mean normalised mRNA expression in the 12 other ovarian cancer cell lines from targeted RNA-Seq data was compared (Figure 3B). The mRNA expression of all 11 genes was fairly similar between the NIH-OVCAR3 cells and the 12 other cell lines. However, protein expression analysis by western blot (Figure 3C) identified more variation at the protein levels between the NIH-OVCAR3 cells and the 12 cell lines. Contrary to the sensitisation to ATRi in cells with low ATM, XRCC1 and XRCC6 expression reported previously in the literature [29,30], the relatively resistant NIH-OVCAR3 cells had lower levels of ATM (3.3-fold), XRCC1 (2.5-fold), and XRCC6 (Ku70) (2.1-fold) compared to the 12 cell lines. Also, despite genomic amplification in ATR, NIH-OVCAR3 cells showed only marginally higher expression (1.4-fold) as compared to the other cell lines. These observations highlight that single gene studies may not be able to identify appropriate determinants of drug response due to the complex genotypic and phenotypic alterations in cancers.

### 2.4. NIH-OVCAR3 Cells Display a Complex HRR Phenotype and Show High Level of NHEJ Activity

**Figure 4:**
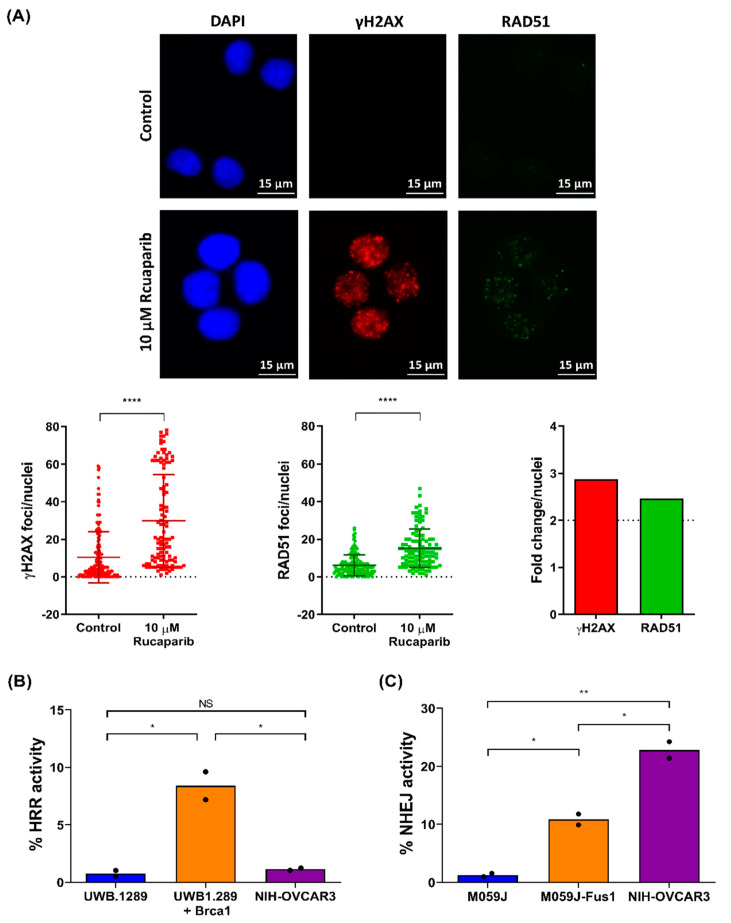
Assessment of HRR and NHEJ in the NIH-OVCAR3 cells. (**A**) Cells were exposed to 0.5% DMSO (control) or 10 µM rucaparib for 48 h before being fixed and stained as per the protocol outlined in the methods. After imaging, the number of γH2AX and RAD51 foci per nucleus was calculated using ImageJ software, data points represent number of foci in an individual nucleus. The fold change in number of foci per nuclei between control and 10 µM rucaparib was then calculated. (**B**) Cells were co-transfected with pDRGFP and pCBASceI plasmids and analysed for the presence of GFP-positive cells 48 h post-transfection. % HRR activity was calculated by normalizing to the untransfected control. Graph shows mean of 2 individual experiments. (**C**) Cells were co-transfected with pimEJ5GFP and pCBASceI plasmids and analysed for the presence of GFP-positive cells 48 h post-transfection. % NHEJ activity was calculated by normalizing to the untransfected control. Graph shows mean of two individual experiments. [* *p* < 0.05; ** *p* < 0.005; **** *p* < 0.0001 (unpaired *t*-test)].

Given the sensitivity of the NIH-OVCAR3 cell line to carboplatin and rucaparib despite the lack of mutations in BRCA/other HRR genes, we investigated the functional status of HRR in NIH-OVCAR3 cells. The nuclear-protein filament necessary for strand invasion during HRR is downstream of the most commonly mutated genes in the HRR pathway and can be visualised by the formation of RAD51 foci. PARP inhibitors are synthetically lethal with HRR defects because the collapsed replication fork that results from PARP inhibition cannot be repaired in an HRR defective cell. Following exposure to 10 µM rucaparib, we observed an induction in γH2AX foci, confirming collapsed replication forks, and an accompanying increase in RAD51 foci formation, indicative of functional HRR (Figure 4A). Thus, both genomic analysis and RAD51 focus formation indicated no loss of HRR function, which could not explain the sensitivity to carboplatin and rucaparib. We therefore further investigated HRR function using a plasmid re-joining assay measured by flow cytometry where the presence of GFP-positive cells indicates effective plasmid re-joining by HRR. Using this method, the NIH-OVCAR3 cell lines showed a low level of HRR activity, similar to that observed in UWB1.289 cells lacking *BRCA1* function, thus confirming HRR deficiency (Figure 4B). The cells ability to form RAD51 foci in response to DNA damage but lack of effective plasmid re-joining by HRR indicates that the HRR defect present in the NIH-OVCAR3 cells is likely downstream of RAD51. NHEJ is another double strand break repair pathway that functions in parallel to HRR. Given the effective loss of function of HRR in the NIH-OVCAR3 cells it was predicted that the cells may have an increased NHEJ activity to compensate for this. Therefore, the level of NHEJ activity in the NIH-OVCAR3 cells was assessed and compared to matched cell lines M059J (lacking DNA-PKcs) and M059J-Fus1 (transfected with a portion of chromosome 8 carrying the DNA-PKcs gene) for NHEJ function (Figure 4C) using flow cytometry based plasmid re-joining assay, where presence of GFP-positive cells indicates plasmid re-joining by NHEJ.

The level of NHEJ activity observed in the NIH-OVCAR3 cells far exceeded that in the M059J-Fus1 cells, despite low Ku70/Ku80 expression and comparable DNA-PK levels to other cell lines. Since previous studies from our lab had identified NHEJ-deficient M059J cells to be 20-fold more sensitive to doxorubicin than the NHEJ-proficient M059J-Fus1 cells [31], we further evaluated if the high NHEJ activity in NIH-OVCAR3 cells also caused resistance to doxorubicin. Interestingly, the percentage cell survival at 100 nM of doxorubicin for NIH-OVCAR3 (~17%) was lower than that previously reported for the M059J-Fus1 cells (~30%) [31] and only 1.4-fold higher than the mean percentage cell survival of the 12 ovarian cancer cell lines (~12%) (Table S2). This again highlights that observations in isogenic pairs of cell lines may not always justify the spectrum of sensitivities observed in a panel of cell lines.

### 2.5. Genomic and Transcriptomic Alterations of DDR Genes among High Grade Serous Ovarian Cancers

To explore the clinical relevance of the genomic or expression level changes in the DDR genes observed in NIH-OVCAR3 cells, we explored the genomic/transcriptomic profile of 38 DDR genes among 201 HGSOCs from the TCGA Pan-cancer data. Of the selected DDR genes 12 had shown genomic amplifications and 19 had shown deep deletions in the NIH-OVCAR3 cells. The remaining seven DDR genes were those whose expression levels (mRNA and protein) were analysed as likely determinants of response to ATRi.

**Figure 5:**
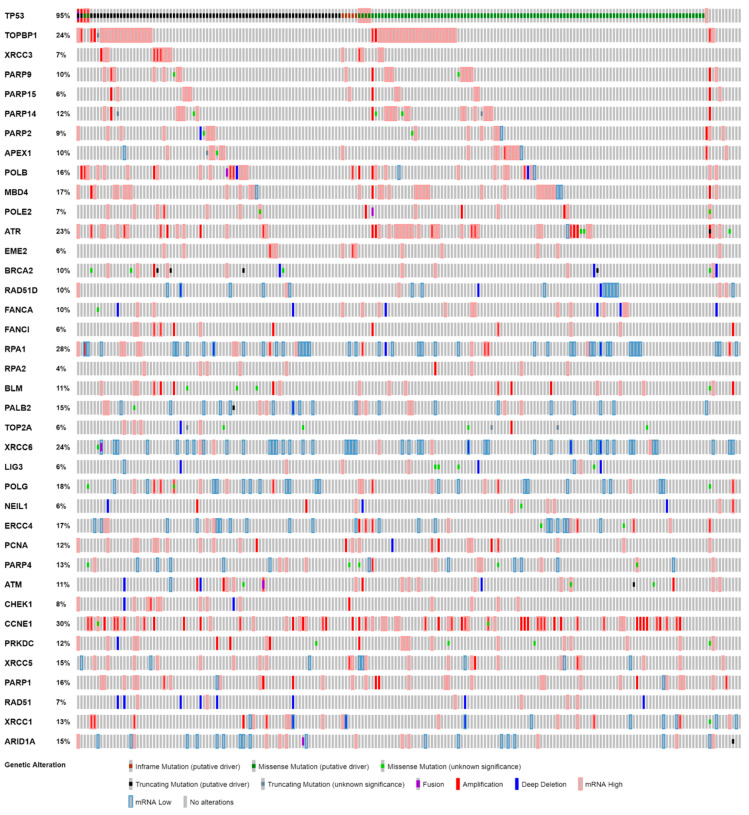
Distribution of genomic and transcriptomic alterations of select DDR genes among 201 HGSOCs. Alteration frequencies of TP53 and DDR genes with amplification and deep deletion in NIH-OVCAR3 cell lines and additional DDR genes analysed as likely determinants of ATRi resistance in NIH-OVCAR3 cell line. Figure generated using cBioPortal (https://www.cbioportal.org/).

Genomic/transcriptomic analysis of genes with genomic amplifications in NIH-OVCAR3 in the TCGA dataset identified that all of the 12 genes are also frequently amplified in HGSOCs, which again emphasizes the similarity of the cell line with HGSOCs. The most amplified DDR genes were TOPBP1 (24%) and ATR (21%). However, analysis of the 19 DDR genes with deep deletions in NIH-OVCAR3 cells, showed that deep deletions in these genes are relatively infrequent clinically in HGSOCs and are more often found to show altered (increased/decreased) expression. Hence, deep deletions in these genes are unlikely to be suitable markers of therapy response in HGSOCs (Figure 5).

Analysis of DDR protein levels (Figure 3C) had identified reduced expression of ATM, XRCC1 and XRCC6 (and marginally increased expression of ATR and RAD51) in NIH-OVCAR3 cells, which was explored further in HGSOCs. It was observed that as opposed to the NIH-OVCAR3 cells, ATM was more likely to be amplified or show higher mRNA expression in ovarian cancers (8%) compared to 2% with deep deletions (or low mRNA levels). XRCC1, on the other hand, showed similar frequencies of amplification (or high mRNA) and deletions (or low mRNA) at 8% and 5%, respectively. Like XRCC1, RAD51 also showed both deep deletions and high mRNA expression at similar frequencies of 4 and 3%, respectively. The two DDR genes that showed similar genomic/transcriptomic alterations as NIH-OVCAR3 cells were XRCC6 (Ku70) and ATR. Greater frequency of deletions or low mRNA expression (22%) in XRRC6 (Ku70) and amplifications or high mRNA expression (21%) in ATR were observed among HGSOCs. Frequent alterations were also observed in the remaining DDR genes (CHEK1, CCNE1, PRKDC, PARP1 and ARID1A) among the HGSOCs, which did not show differential protein expression in NIH-OVCAR3 cells (Figure 5).

Platinum sensitivity data was available for 148/201 HGSOCs. We attempted to see if there was any difference in mRNA expression of these genes between the platinum-sensitive and -resistant groups. However, none of the genes showed a statistically significant difference between the two groups (Table S4), except FANCI which showed marginal but statistically significantly reduced expression in the platinum-resistant subgroup, which was not in line with the platinum and PARPi sensitivity of NIH-OVCAR3. This again highlights the significance of analysing both protein expression and function in relation to therapy response, where genomic/transcriptomic alterations fail to show any association with chemotherapy response.

## 3. Discussion

Cell lines derived from human cancers are extensively used models of diseases in preclinical research. Recent advances in the genomic and transcriptomic characterisation of cell lines has greatly expanded their applicability in exploring drug response. Although often representative of the corresponding cancer type, like individual tumours, cell lines can demonstrate variable drug response profiles due to the complex genomic characteristics and functional activity of important cellular pathways. While being representative of HGSOCs, with functional loss of TP53 and high frequency of CNAs, we found the NIH-OVCAR3 cell line to be an outlier with a complex drug sensitivity profile. The cells were found to be resistant to ATRi, and very sensitive to platinum agents (carboplatin and cisplatin) and PARPi, rucaparib, which prompted exploration of likely alterations in DDR pathways. However, this response could not be predicted by genomic or proteomic characterisation of DDR pathways alone, highlighting the need for functional characterisation in combination with genomics to better predict drug response.

Inactivation of p53 is one of the most well studied determinants of sensitivity to ATR inhibition. However, despite the loss of p53 function, the NIH-OVCAR3 cells were resistant to VE-821 and this resistance was not due to inadequate ATR inhibition, which was consistent with that observed in other ovarian cancer cell lines. This highlights that functional loss of p53 may not always cause sensitivity to cell cycle checkpoint inhibition by targeting ATR. This rationale further gains strength from conflicting reports of response to ATRi in different p53 mutant models. In previous reports the ATRi, AZD6738, has been found to be selectively cytotoxic towards p53 mutant CLL cells [32]. However, sensitivity to AZD6738 was found to be independent of p53 status in a panel of cell lines, including the HCT116 p53 isogenic pair [33]. Furthermore, Middleton et al. (2018), reported no difference in sensitivity to single agent VE-821 in the same HCT116 p53 isogenic cell line pair [34]. In our study, gene expression analysis identified more than 2-fold lower expression of 3 proteins (ATM, XRCC1 and Ku70) in the NIH-OVCAR3 cells compared to the 12 other cell lines. This again is in contrast to observations in the literature, where loss of ATM, XRCC1 and XRCC6 (Ku70) has been reported to confer ATRi sensitivity when each gene is investigated in isolation [15,27,29,30,32,35,36]. As discussed above, this highlights the difficulty in translating determinants of sensitivity identified in knockdown studies investigating a single gene at a time or in isogenic cell pairs, into the context of a panel of cell lines and tumour samples with heterogeneous genetic and functional characteristics.

Interestingly, NIH OVCAR3 cells had amplifications in ATR and TOPBP1 genes. Downregulation of these genes has previously been reported to confer sensitivity to VE-821 [37,38] and hence amplification of these genes could contribute to the resistance to VE-821 observed in the NIH-OVCAR3 cells. TOPBP1 is an established activator of the ATR signalling and has also been identified as a synthetic lethal target for ATRi sensitivity [37], which demands further exploration for its likely role in ATRi resistance. While several genes showing alterations in NIH-OVCAR3 cells were also frequently altered in HGSOCs, the genomic/expression level alterations in ATR, TOPBP1 and XRCC6 (Ku70) are likely to be more clinically relevant as determinants of resistance/sensitivity to ATRi, given these alterations observed in the NIH-OVCAR3 cells are also very common among HGSOCs (frequencies > 20%).

As opposed to the relative resistance to ATRi, the NIH-OVCAR3 cells showed strong sensitivity to carboplatin and PARPi, rucaparib, similar to cell lines with established HRR defects. In the published literature, sensitivity of NIH-OVCAR3 to platinum agents have been reported with conflicting findings, with Beaufort et al. finding the cells to be relatively sensitive to carboplatin [10] whilst the GDSC data ranks NIH-OVCAR3 as the 9th most resistant to cisplatin out of the 25 ovarian cancer cell lines analysed (https://www.cancerrxgene.org/). This, however, can be driven by differences in the methodologies used to assess the sensitivity, which in turn can be dependent on the doubling time of the cell line. In this study, we used the colony formation assay which is expected to be free from interpretation bias resulting from differences in cell doubling time and found the cells to be particularly sensitive to platinum agents and PARPi.

HRR defects are a known determinant of sensitivity to both platinum [39] and PARP inhibitors. However, genomic analysis identified no functionally inactivating mutations of HRR genes. Therefore, the functional HRR ability of the NIH-OVCAR3 cells was assessed. While, the cells demonstrated a clear ability to form RAD51 foci following DNA damage induction, the plasmid re-joining assay identified the cells as HRD, explaining the sensitivity to both platinum agents and rucaparib. The ability of the cells to form RAD51 foci whilst incapable of performing plasmid re-joining by HRR is a novel finding and indicates that the defect is downstream of RAD51, as has been identified in a non-small cell lung cancer cell line, Calu-6, where RAD51 foci fail to resolve leading to their persistence causing incomplete DNA repair [40]. The HRD phenotype of the cells was not evident by mutational profile, however in an analysis performed by Jarvis et al., COSMIC signature 3 was detected in the cells indicating likely HRR defects [41], although the mutational activity of Signature 3 for NIH-OVCAR3 cells (114.3) was reported to be close to the mean mutation activity of 60 ovarian cancer cell lines (151.6) analysed by the CCLE study [42]. This indicates that the mere presence of HRD gene signature may not be a good indicator of HRR status. Moreover, whilst single gene mutation screens (for example, *BRCA*) may have value when looking at larger cohorts, it can limit therapeutic options for some individuals.

Given the loss of HRR, it was unsurprising that the NIH-OVCAR3 cells were found to have a high level of NHEJ activity [43]. However whilst a high level of NHEJ has been previously shown to confer resistance to doxorubicin using isogenic cell line pairs, NIH-OVCAR3 cells showed only marginally high % survival following 100 nM doxorubicin treatment as compared to the other 12 ovarian cancer cell lines. Again, highlighting the fact that response to chemotherapy can be driven by multiple factors.

## 4. Materials and Methods

### 4.1. Cell Line and Culture Conditions

The NIH-OVCAR3 cell line (ATCC HTB-161) was cultured in RPMI supplemented with 10% FBS and incubated at 37 °C in humidified atmosphere containing 5% CO_2_. The growth conditions of additional 12 ovarian cancer cell lines used for comparison, including UWB1.289 and UWB1.289 + BRCA1 cell lines used as positive and negative controls for HRR assay, are listed in Table S4. M059J, DNA-PKcs-deficient human glioblastoma were cultured in DMEM supplemented with 10% FBS and incubated at 37 °C [44]. M059J-Fus-1 (M059J transfected with a portion of chromosome 8 carrying the DNA-PKcs gene) were cultured in full media supplemented with 400 μg/mL G418 [45]. Cell lines were authenticated by Short Tandem Repeat (STR) profiling using the PowerPlex Fusion System (Promega, Southampton, UK). Cell lines were mycoplasma tested every three months.

### 4.2. Chemicals and Reagents

The chemotherapy drugs used for the cytotoxicity assay include Carboplatin (Sigma Aldrich, St. Louis, MO, USA), rucaparib and VE-821 (kind gifts from Pfizer Global R & D, Boulder, CO, USAand Merck KGaA, Darmstadt, Gemanyrespectively). Drugs used for western blot analysis include Nutlin-3 (Tocris, Bristol, UK) and 4NQO (Sigma Aldrich).

### 4.3. Colony Formation Assay

Cells were plated into 6-well tissue culture dishes at low densities and allowed to adhere for 24 h before being treated for 24 h with carboplatin (0–50 µM) or rucaparib (0–30 µM), or 48 h with VE-821 (0–30 µM). DMSO concentration was kept constant at 0.5%, including no-drug control, for drugs dissolved in DMSO. Following treatment, the medium was removed and replaced with fresh drug-free medium to allow colony formation. The cells were fixed in methanol: acetic acid (3:1 *v*/*v*) and stained with 0.4% crystal violet. Colonies were counted and the % survival for each treatment was calculated from the relative plating efficiency of treated versus untreated controls.

### 4.4. Homologous Recombination Repair (HRR) Assay

#### 4.4.1. Immunofluorescence Based γH2AX-RAD51 Assay

HRR was assessed by immunofluorescence. Cells were treated for 48 h with 10 μM rucaparib with 0.5% DMSO concentration for the untreated and treated cells. To assess DNA damage and functional repair, cells were stained with mouse monoclonal anti phospho-histone H2A.X (Ser139: γH2AX) antibody (Upstate/Millipore, Burlington, NJ, USA) at 1:1000 and rabbit monoclonal anti RAD51 antibody (AbCam, Cambridge, UK) at 1:500. Secondary antibodies used were Alexa 488 conjugated goat anti-rabbit and Alexa 546 conjugated goat anti-mouse (Invitrogen, Waltham, MA, USA), both at 1:1000. The nuclei were stained with DAPI. The number of γH2AX and RAD51 foci in each cell was quantified using ImageJ software and data was plotted using GraphPad Prism (GraphPad, San Diego, CA, USA). A >2-fold increase in γH2AX foci formation was used as an indicator of DNA damage induction following rucaparib treatment and a >2-fold increase in RAD51 foci formation was indicative of functional HRR.

#### 4.4.2. Plasmid Re-Joining Assay

pDRGFP (plasmid # 26475, Addgene, Watertown, MA, USA) and pCBASceI (plasmid # 26477, Addgene) plasmids used for the HRR assay were a kind gift from Maria Jasin [46,47]. Equimolar quantities of both plasmids were used for co-transfection of the cells using Lipofectamine 3000 reagent according to the manufacturer’s instructions. The cells were collected by trypsinization, washed in PBS then re-suspended in 500 µL of PBS. A total of 50,000 cells were analysed by flow cytometry to estimate the number of GFP-positive cells. The % HRR activity was calculated by subtracting the % of GFP-positive cells in untransfected from the co-transfected sets.

### 4.5. Non-Homologous End Joining (NHEJ) Plasmid Re-Joining Assay

pCBASceI (Addgene plasmid # 26477) and pimEJ5GFP (plasmid # 44026, Addgene) plasmids used for the NHEJ assay were a kind gifts from Maria Jasin and Jeremy Stark [47,48]. Equimolar quantities of both plasmids were used for co-transfection of the cells and the same method as detailed for the HRR assay was used to estimate the % NHEJ activity.

### 4.6. Western Blot Analysis

Whole cell lysates were prepared from exponentially growing cells in Phosphosafe extraction reagent buffer (Merck Millipore, Burlington, MA, USA) containing 1:100 protease inhibitor cocktail (Thermo Fisher Scientific, Waltham, MA, USA). The protein content of all lysates was determined by Pierce BCA assay (Thermo Fisher Scientific) following manufacturers protocol. Lysates were diluted to equal concentrations between 0.5–1 mg/mL in 2 × Laemmli buffer (BioRad, Hercules, CA, USA) for TP53 functional analysis, or XT sample buffer and XT reducing agent (BioRad) for VE-821 inhibition and baseline protein expression analysis, and boiled for 5 min at 95 °C. Gel electrophoresis was performed using 4–20% criterion TGX polyacrylamide gels for the TP53 functional analysis, and 3–8% XT Tris-Acetate gels for the VE-821 inhibition and baseline protein expression for optimum separation based on the molecular weights of the proteins analysed. Proteins were transferred to nitrocellulose Hybond™ C membranes (Amersham, Buckinghamshire, UK) and blocked with 5% milk (*w*/*v*) in TBS-T for at least 1 hr before primary antibodies were added as indicated for each western below, diluted in either 5% milk (*w*/*v*) or 5% BSA (*w*/*v*) in TBS-T. Clarity Western enhanced chemiluminescence substrate (Bio-Rad) was used to visualize the bands following the manufacturer’s protocol, and chemiluminescence measured using G-box gel documentation system (Syngene, Cambridge, UK).

#### 4.6.1. TP53 Functional Analysis

Cells were treated with Nutlin-3 at indicated doses for 4 h before cells lysates were harvested. Primary antibodies: mouse anti-MDM2 (1:300, # OP46-100UG, Merck Millipore), mouse anti-p21^WAF1^ 1:100 (#: OP64, Merck Millipore), mouse anti-p53 (1:500, # NCL-L-p53-DO7, Leica Microsystems Ltd., Newcastle upon Tyne, UK) and mouse anti-α-tubulin (1:20,000, # T6074, Sigma) as a loading control (Figure S2).

#### 4.6.2. VE-821 ATR Inhibition Analysis

Cells were treated with 4NQO ± VE-821 at the indicated doses for 1 h before cell lysates were harvested. Primary antibodies: goat anti-ATR (1:500, # 1887, Santa Cruz, Dallas, TX, USA), rabbit anti-CHK1 (Ser345) (1:1000, # 2341, Cell Signalling, Cell Signalling, Danvers, MA, USA) and rabbit anti-vinculin (1:1000, # 4650, Cell Signalling).

#### 4.6.3. Antibodies Used for Baseline Protein Expression Analysis

Cells were left to reach 70–80% confluence before cell lysates were harvested. Primary antibodies: rabbit anti-ATM (1:500, #2873, Cell Signalling), goat anti-ATR (1:500, # 1887, Santa Cruz), mouse anti-CHK1 (1:500, # 8408, Santa Cruz), mouse anti-Cyclin E (1:500, # 247, Santa Cruz), rabbit anti-DNA-PKcs (1:500, # 9051, Santa Cruz), mouse anti-Ku70 (1:500, #3114, Abcam), rabbit anti-Ku80 (1:500, #80592, Abcam), rabbit anti-PARP-1 (1:500, # 227244, Abcam), rabbit anti-RAD51 (1:500, # 8349, Santa Cruz), rabbit anti-XRCC1 (1:500, #11429, Santa Cruz), rabbit anti-ARID1A (1:1000, #12354, Cell Signalling). Ponceau-S staining was used to ensure equal loading of the protein.

### 4.7. Targeted RNA-Sequencing of the Cell Line Panel

Cell pellets containing ~40,000 cells were collected of the 13 ovarian cancer cell lines (Table S3) including the NIH-OVCAR3 cells, and cryo-preserved and shipped for targeted RNA-Sequencing analysis by HTG Molecular Diagnostics Inc. (Tucson, AZ, USA). The samples were run on the Illumina NextSeq (Illumina, San Diego, CA, USA) with the HTG EdgeSeq Oncology biomarker panel assay (961–002) containing probes against 2567 genes including 15 housekeeper genes, and four negative and four positive process control genes. The HTG EdgeSeq Parser was used to align FASTQ files to probe list by the company. Following quality control, the raw read counts were estimated followed by estimation of read counts per million after median normalisation. The normalized gene expression for selected DDR genes was calculated by using expression of ACTB gene as the normalization control, which showed the least standard deviation of gene expression across cell lines. The data is accessible through GEO submission GSE150942 (https://www.ncbi.nlm.nih.gov/geo/query/acc.cgi?acc=GSE150942).

### 4.8. Bioinformatics Analysis

RNA-Seq data (read counts) for NIH-OVCAR3 cell line was downloaded from the CCLE database (https://portals.broadinstitute.org/ccle/data). To confirm the authenticity of the cell line used in this study, RNA-Seq read counts for 2544 genes were extracted and the genes with no data availability or no expression were excluded. The gene expression data of 2426 genes was used to calculate the Pearson’s correlation co-efficient and corresponding *p*-value between the two datasets. The copy number alteration and mutation data for NIH-OVCAR3 cell line was also retrieved from the CCLE database. The mutations were categorised as homozygous or heterozygous mutation using a variant allele frequency cut-off of 0.8, with mutations with variant allele frequency >0.8 were considered as homozygous mutations. The copy number alterations were categorised using their GISTIC scores with −2 for deep deletion and 2 for amplification. The mutation and copy number alteration data of the CCLE dataset was accessed using cBioportal web tool (https://www.cbioportal.org/) [24,49] Additionally, data on 201 HGSOCs was also analysed for copy number alterations, mutations and gene expression. For a subset of cancers, the data for platinum response could be retrieved and was used to identify any differences in gene expression between the platinum sensitive and resistant subgroups.

### 4.9. Statistical Analysis

All statistical analysis was performed using GraphPad Prism 8.

## 5. Conclusions

In conclusion, the data presented here highlight the difficulties in predicting sensitivity to emerging therapeutics. Even with extensive genomic characterisation of cell lines, determinants of sensitivity to therapeutics investigated in isolation in isogenic matched cell lines often fail to translate to the context of cell lines with complex molecular characteristics that mirror individual tumours. The loss of p53 and decreased expression of ATM, XRCC1 and XRCC6 (Ku70) would predict sensitivity to ATRi VE-821. However, the NIH-OVCAR3 cells were resistant to VE-821. Loss of p53 and ATM, as determinants of ATRi response, are also being investigated in numerous clinical trials as outcome measures (NCT03641313, NCT02157792, NCT03718091, NCT02264678, NCT01955668 and NCT03896503), and will be interesting in relation to the observations made here in NIH-OVCAR3 cells. Furthermore, mutational characterisation did not predict the profound sensitivity of NIH-OVCAR3 cells to carboplatin and rucaparib and even functional analysis using RAD51 foci indicated its limitations. These findings have parallels in the clinic where although *BRCA* mutational status and platinum sensitivity are good indicators of likely PARPi response they are by no means 100% predictive of response [50]. Thus, the need for a combinatorial approach for molecular and functional characterization of individual cancers appears to be an important strategy for the development of personalized medicine approaches in cancer therapy.

## 6. Patents

1. Helleday, T.; Curtin NJ. Therapeutic Compounds (PARP inhibitors in homologous repair/BRCA defective cancer) WO 2005/012305 A2.
2. Boritzki, T.J.; Calvert, A.H.; Curtin, N.J.; Dewji, M.R.; Hostomsky, Z.; Jones, C.; Kaufman, R.; Klamerus, K.J.; Newell, D.R.; Plummer, E.R.; Reich, S.D.; Steinfeldt, H.M.; Stratford, I.J.; Thomas, H.R.; Williams, K.J. Therapeutic Combinations Comprising PARP inhibitor WO/2006/033006.
3. Falcon, S.; Reaper, P.; Pollard, J.; Curtin, N.J.; Middleton, F.K.; Chen, T. Method for measuring ATR inhibition mediated increases in DNA damage. WO2014055756A1.

## Supplementary Materials

The following are available online at https://www.mdpi.com/2072-6694/12/7/1939/s1, Figure S1: Sensitivity of NIH-OVCAR3 cells to Cisplatin, Figure S2: Confirmation of loss of TP53 function in NIH-OVCAR3 cells, Figure S3: Western blot confirming the loss of TP53 function in NIH-OVCAR3 cells, Figure S4: Western blot of ATR activation and inhibition by 4NQO ± VE-821 in NIH-OVCAR3 cells, Figure S5: Western blot of baseline protein expression in panel of ovarian cancer cell lines, Table S1: Cisplatin sensitivity, Table S2: Additional drug sensitivity, Table S3: Culture medium of additional 12 ovarian cancer cell lines used for comparison to the NIH-OVCAR3 cell line, Table S4: mRNA expression in platinum-sensitive and resistant groups.
