# Supplementary material for: Characterisation of Ovarian Cancer Cell Line NIH-OVCAR3 and Implications of Genomic, Transcriptomic, Proteomic and Functional DNA Damage Response Biomarkers for Therapeutic Targeting
Source: Cancers (Basel). 2020 Jul 17;12(7):1939. doi: 10.3390/cancers12071939 (PMC7409137; doi:10.3390/cancers12071939)
Supplement: Supplementary file 1 [file cancers-12-01939-s001.pdf]

Supplementary Materials

Characterisation of Ovarian Cancer Cell Line NIH-OVCAR3 and Implications of Genomic, Transcriptomic, Proteomic and Functional DNA Damage Response Biomarkers for Therapeutic Targeting

Alice Bradbury, Rachel O'Donnell, Yvette Drew, Nicola J Curtin and Sweta Sharma Saha

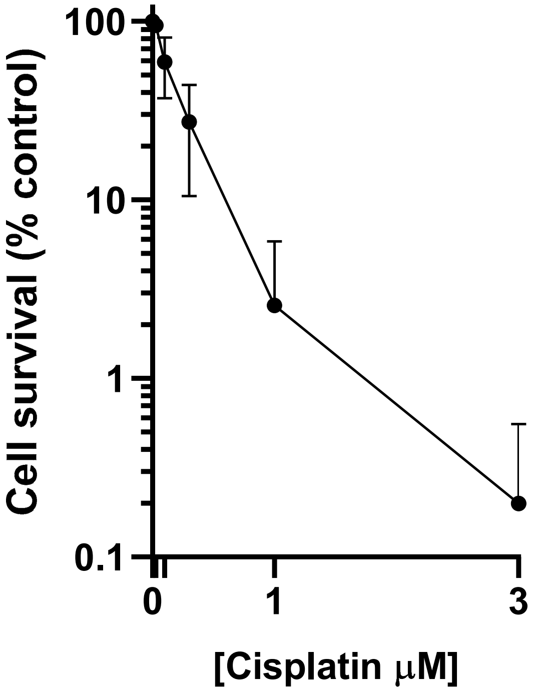

Figure S1. Sensitivity of NIH-OVCAR3 cells to Cisplatin. NIH-OVCAR3 cells were exposed to Cisplatin for 48 hrs. Media was replaced with drug free media for 21 days to allow colony formation. Data are the mean and standard deviation of at least three independent experiments.

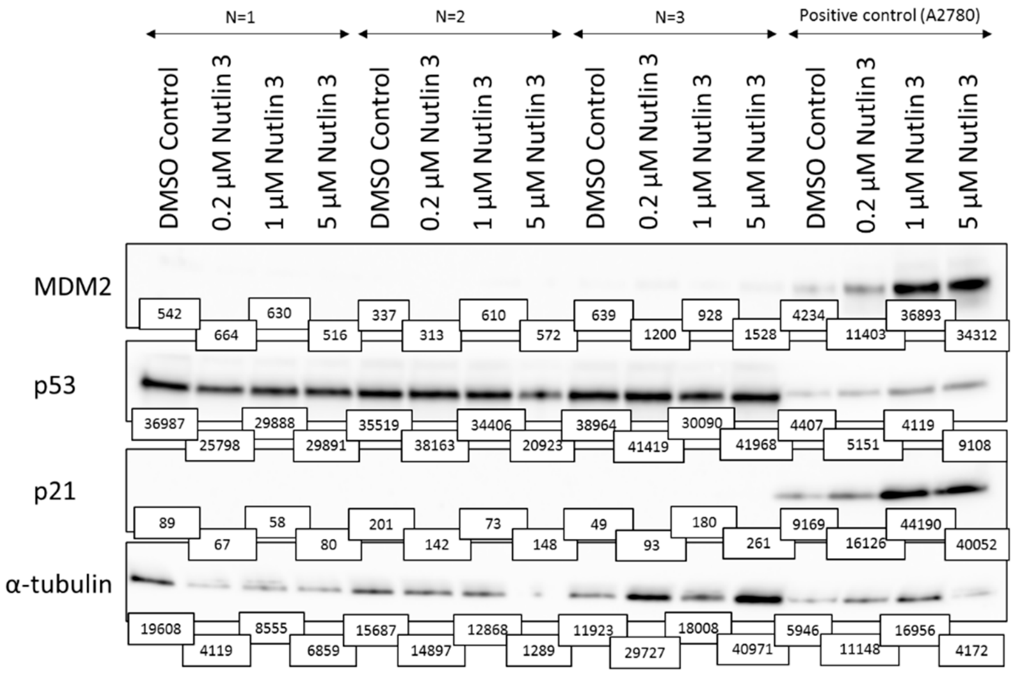

Figure S2. Confirmation of loss of TP53 function in NIH-OVCAR3 cells. NIH-OVCAR3 and A2780 cells were exposed to Nutlin-3 at indicated concentrations for 4 hrs before western blotting. The blots were cut at appropriate molecular weight markers before chemiluminescent detection of protein bands.

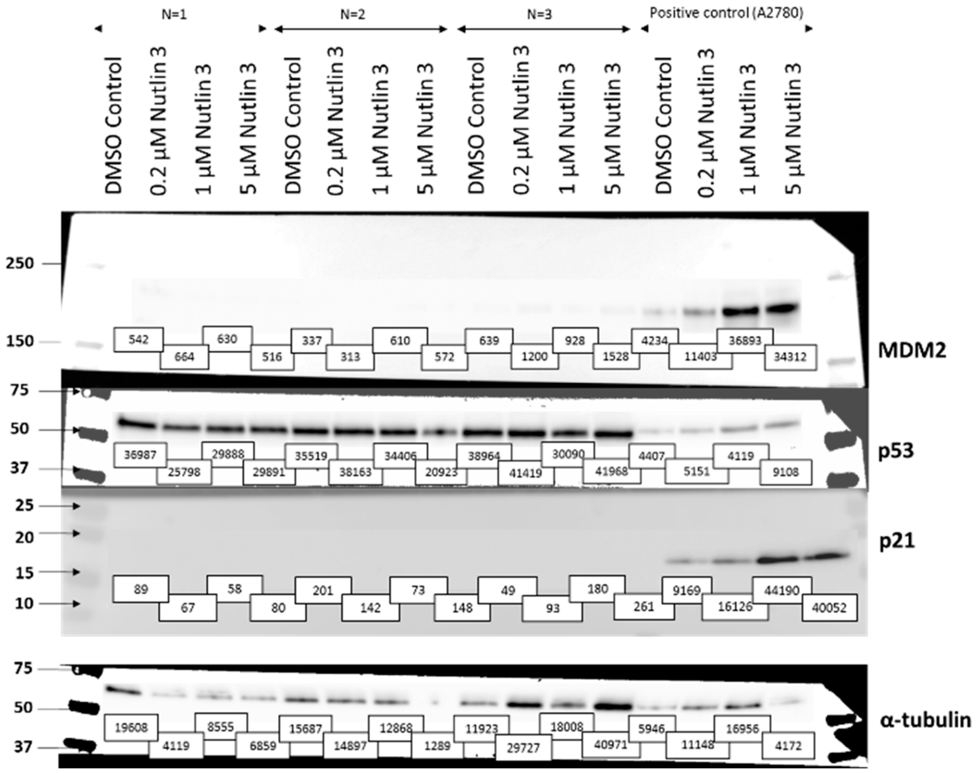

Figure S3. Figure 2 (Original western blot images): Western blot confirming the loss of TP53 function in NIH-OVCAR3 cells. Densitometry calculated using ImageJ software, value given is AUC – background to 1 decimal place. The blots were cut at appropriate molecular weight markers before chemiluminescent detection of protein bands.

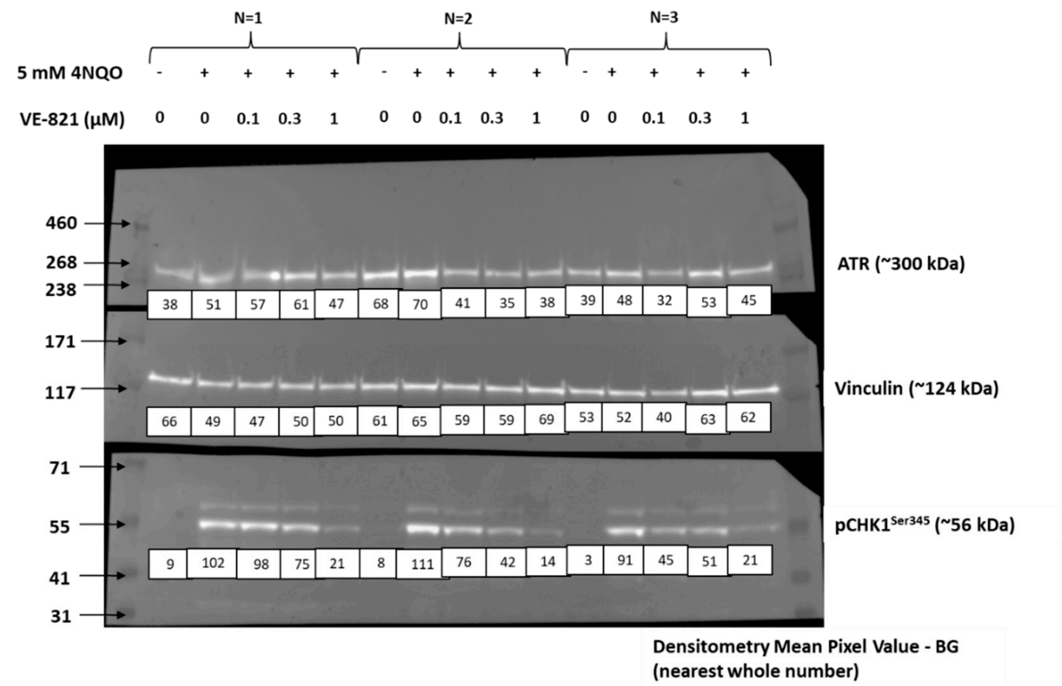

Figure S4. Figure 3A (Original western blot images): Western blot of ATR activation and inhibition by 4NQO ± VE-821 in NIH-OVCAR3 cells. Densitometry value to nearest whole number given under each band. The blots were cut at appropriate molecular weight markers before chemiluminescent detection of protein bands.

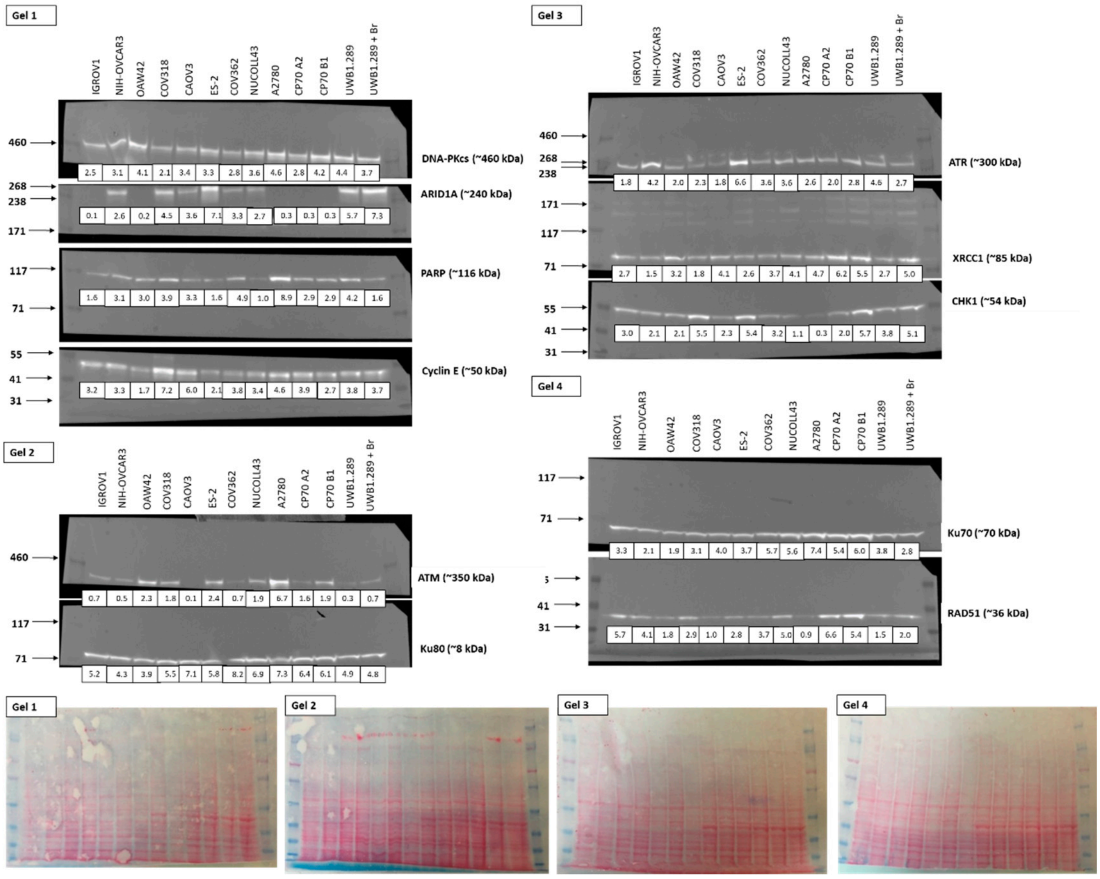

Figure S5. Figure 3C (Original western blot images): Western blot of baseline protein expression in panel of ovarian cancer cell lines. Densitometry calculated using ImageJ software, value given is AUC – background to 1 decimal place. The blots were cut at appropriate molecular weight markers before chemiluminescent detection of protein bands.

Table S1. Cisplatin sensitivity**.** Cells were exposed to cisplatin for 48 hrs then medium was replaced with drug free medium for up to 21 days to allow colony formation. Data are the mean and standard deviation of at least three independent experiments in NIH-OVCAR3 cells and the mean and standard deviation of two comparative ovarian cancer cell lines.

Cell Line	Cisplatin LC_{50} ± SD (μM)
**NIH-OVCAR3**	0.18 ± 0.08
**2 ovarian cancer cell lines**	0.70 ± 0.32

Table S2. Additional drug sensitivity. Cells were exposed to doxorubicin, gemcitabine, paclitaxel or Table 24. hrs then medium was replaced with drug free medium for up to 21 days to allow colony formation. Data are the mean and standard deviation of at least three independent experiments in NIH-OVCAR3 cells and the mean and standard deviation of 12 comparative ovarian cancer cell lines.

Cell Line	Doxorubicin LC_{50} ± SD (nM)	Gemcitabine LC_{50} ± SD (nM)	Topotecan LC_{50} ± SD (nM)	Paclitaxel LC_{50} ± SD (nM)
**NIH-OVCAR3**	55.03 ± 1.97	41.55 ± 1.2	7.2 ± 0.36	2.71 ± 0.08
**12 ovarian cancer cell lines**	44.46 ± 15.54	28. 69± 27.92	15.6 ± 9.32	3.37 ± 2.41

Table S3. Culture medium of additional 12 ovarian cancer cell lines used for comparison to the NIH-OVCAR3 cell line.

Cell lines	Culture Media
**COV362**	DMEM, 10% FBS
**COV318**	DMEM, 10% FBS
**CAOV3**	DMEM, 10% FBS
**ES2**	RPMI 1640, 10 % FBS
**OAW42**	RPMI 1640, 10 % FBS
**A2780**	RPMI 1640, 10 % FBS
**CP70-B1**	RPMI 1640, 10 % FBS + 200 μg/ml Hygromycin B
**CP70-A2**	RPMI 1640, 10 % FBS + 200 μg/ml Hygromycin B
**IGROV1**	RPMI 1640, 10 % FBS
**UWB1.289**	50% RPMI-1640 + 50% MEGM Bullet Kit medium
**UWB1.289+BRCA1**	50% RPMI-1640 + 50% MEGM Bullet Kit medium + 200 μg/ml G-418
**NUCOLL43**	RPMI 1640, 20 % FBS

Table S4. mRNA expression in platinum-sensitive and –resistant groups. Mean log-transformed RSEM values of 37 genes (gene expression data for RAD51D was not available) in the platinum sensitive and resistant subgroups from the TCGA pan-cancer cohort and the corresponding p-value for estimation of any statistically significant difference.

Gene name	Platinum-sensitive (Mean log2RSEM values)	Platinum-resistant (Mean log2RSEM values)	*p*-value
ATM	9.33	9.38	0.65
ATR	9.23	9.21	0.69
CHEK1	8.52	8.35	0.45
CCNE1	9.36	9.41	0.69
PRKDC	12.02	11.85	0.29
XRCC5	13.07	13.09	0.77
XRCC6	12.6	12.48	0.18
PARP1	12.26	12.12	0.19
RAD51	8.2	8.2	0.9
XRCC1	9.85	9.81	0.81
ARID1A	11.47	11.52	0.32
TOPBP1	10.2	10.24	0.63
XRCC3	8.67	8.5	0.39
PARP9	11.09	10.83	0.18
PARP14	11.46	11.15	0.11
PARP15	2.95	2.78	0.54
PARP2	9.48	9.27	0.15
APEX1	12.48	12.4	0.94
POLB	8.78	8.95	0.25
MBD4	10.03	10.06	0.92
EME2	3.49	3.52	0.69
POLE2	6.58	6.39	0.23
TP53	10.88	11.07	0.36
BRCA2	6.65	6.58	0.72
FANCA	8.48	8.31	0.32
FANCI	*9.87*	*9.56*	*0.02**
RPA1	11.21	11.26	0.43
RPA2	10.4	10.57	0.17
BLM	7.59	7.39	0.34
PALB2	8.38	8.36	0.59
TOP2A	11.11	11.17	0.58
PARP4	10.92	10.97	0.5
LIG3	9.19	9.12	0.53
POLG	9.72	9.49	0.06
NEIL1	6.06	5.95	0.5
ERCC4	7.94	8.11	0.39
PCNA	11.7	11.69	0.79

© 2020 by the authors. Licensee MDPI, Basel, Switzerland. This article is an open access article distributed under the terms and conditions of the Creative Commons Attribution (CC BY) license (http://creativecommons.org/licenses/by/4.0/).
